# Supplementary material for: Genome-Wide Identification and Expression Analysis of XTH Gene Family during Flower-Opening Stages in Osmanthus fragrans
Source: Plants (Basel). 2022 Apr 8;11(8):1015. doi: 10.3390/plants11081015 (PMC9031776; doi:10.3390/plants11081015)
Supplement: Supplementary file 1 [file plants-11-01015-s001.zip › Figure S1.pdf]

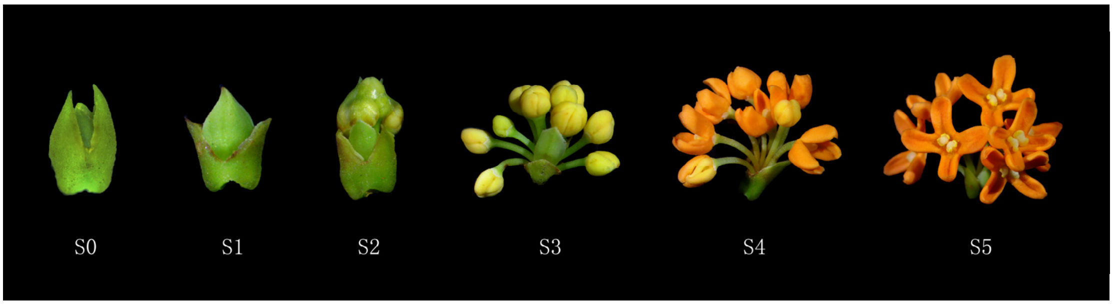

Figure S1. Flower bud phenotype of O. fragrans at different stages. S0, the outer bud scales unfurled and the inner bud scales still furled; S1, the bud became globular-shaped and the inside bracts covering the inflorescence was visible; S2, the inflorescence burst through bracts and the florets closely crowded; S3, the florets are bud shaped and the pedicels elongate; S4, initial flowering stage; S5, full flowering stage.
