# Supplementary material for: Genome-Wide Identification and Expression Analysis of XTH Gene Family during Flower-Opening Stages in Osmanthus fragrans
Source: Plants (Basel). 2022 Apr 8;11(8):1015. doi: 10.3390/plants11081015 (PMC9031776; doi:10.3390/plants11081015)
Supplement: Supplementary file 1 [file plants-11-01015-s001.zip › Figure S2.pdf]

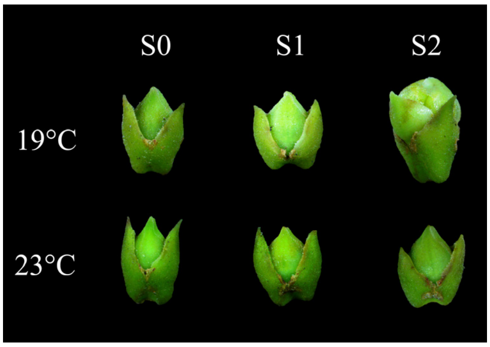

Figure S2. Flower bud phenotype under temperature treatment. When flower buds treated at 19° C reached S1 and S2 period, the buds treated at 23° C still stayed at S1 period all the time.
